# Trigeminal Nerve Stimulation for Disorders of Consciousness: A Narrative Review of Potential Mechanisms, Clinical Evidence, and Future Perspectives

**DOI:** 10.1002/cns.71148

**Published:** 2026-10-20

**Authors:** Haodong Niu, Yilin Zhang, Nan Wang, Xiaoke Chai, Yiyi Zhai, Zhiyao Zheng, Yangxi Xu, Yitong Jia, Kefei Wan, Sipeng Zhu, Tianqing Cao, Qiheng He, Minghao Liu, Zhuoyang Li, Yichen Wang, Haitao Huang, Juanning Si, Yi Yang, Jizong Zhao

**Affiliations:** ^1^ Department of Neurosurgery Beijing Tiantan Hospital, Capital Medical University Beijing China; ^2^ School of Instrumentation Science and Opto‐Electronics Engineering, Beijing Information Science and Technology University Beijing China; ^3^ Department of Neurosurgery The People's Hospital of Liaoning Province Liaoning China; ^4^ Department of Neurosurgery Aviation General Hospital Beijing China; ^5^ Department of Neurosurgery The First Hospital of Hebei Medical University Shijiazhuang China

**Keywords:** arousal circuits, clinical trial, disorders of consciousness, neuromodulation, trigeminal nerve stimulation

## Abstract

**Background:**

Disorders of consciousness (DoC), including the vegetative state/unresponsive wakefulness syndrome (VS/UWS) and the minimally conscious state (MCS), present a major clinical challenge due to limited therapeutic options. Trigeminal nerve stimulation (TNS) has recently emerged as a promising neuromodulatory intervention.

**Mechanisms and Current Evidence:**

The trigeminal nuclei send efferent fibers to the thalamus and the ascending reticular system, which then broadly innervate multiple brain areas via the ascending reticular activating system (ARAS) through both direct and indirect pathways. Capitalizing on the trigeminal nerve's distinctive anatomy, TNS has been hypothesized to potentially promote consciousness recovery in preclinical models by activating specific pathways, modulating the release of key neurotransmitters (e.g., hypocretin, dopamine, and norepinephrine), and influencing cerebral hemodynamics. Furthermore, randomized controlled trials (RCTs) involving both external TNS (eTNS) and acoustic‐electric TNS have highlighted a possible direction for its application as a novel therapy for DoC; the findings appear to suggest potential clinical relevance, although current evidence remains constrained by small samples, patient heterogeneity, variable stimulation protocols, and limited long‐term follow‐up.

**Challenges:**

The clinical translation of TNS, however, faces pressing obstacles, including uncertainty regarding optimal stimulation targets and parameters, insufficient long‐term safety and efficacy data, incompletely elucidated mechanisms of action, and a lack of suitable preclinical models.

**Conclusions:**

TNS may represent a promising therapeutic approach for DoC, but further high‐quality studies are needed to establish its mechanisms, safety, efficacy, and optimal stimulation strategies.

## Graphical abstract

Preclinical studies indicate that Trigeminal Nerve Stimulation (TNS) treats Disorders of Consciousness (DoC) by activating neural pathways that promote the release of orexin, dopamine, and norepinephrine. Additionally, TNS modulates cerebral hemodynamics and immunoregulation to enhance consciousness in experimental animals. Clinical trials show significant improvements in Glasgow Coma Scale (GCS) and Coma Recovery Scale‐Revised (CRS‐R) scores in the TNS group, with more patients achieving or emerging from Minimally Conscious State (MCS) compared to the sham stimulation group. Subgroup analyses reveal greater efficacy in patients with Vegetative State (VS), shorter disease duration, and etiology due to traumatic brain injury (TBI) or stroke, highlighting the potential of multimodal stimulation.

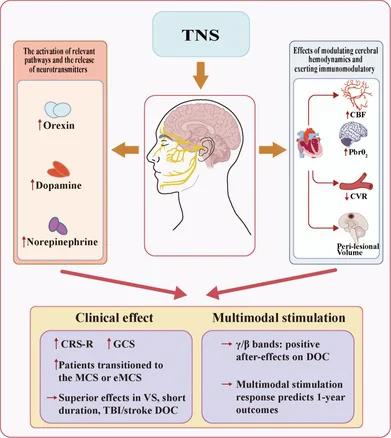

## 1 Introduction

Disorders of consciousness (DoC) refers to states of lost consciousness caused by various severe brain injuries, such as vegetative state/unresponsive wakefulness syndrome (VS/UWS) and minimally conscious state (MCS). Prolonged disorders of consciousness (pDoC) refer to disorders of consciousness in which unconsciousness persists for more than 28 days [1, 2]. The survival period for patients with pDoC is generally 2 to 5 years. Among them, patients in MCS have a better potential for recovery, whereas those in VS face greater difficulty in regaining consciousness. This results in a severe dependence on performing activities of daily living. It has been reported that the lifetime care costs for a patient with pDoC may exceed $1,000,000, imposing a tremendous burden on the patients, their families, and society.

Over the past decades, various neuromodulation strategies have been explored in DoC. Currently, for disorders of consciousness (DoC) patients, most therapies proposed—such as hyperbaric oxygen, nutraceuticals, stem cell therapy, and evening primrose oil—lack sufficient evidence to either support or refute their use, with many also carrying associated risks. The exception to this is amantadine [1]. Recent years have witnessed a growing array of neuromodulation techniques—including Deep Brain Stimulation (DBS) [3, 4], Spinal Cord Stimulation (SCS) [5, 6], Vagus Nerve Stimulation (VNS) [7], Transcranial Direct Current Stimulation (tDCS) [8, 9], repeated Transcranial Magnetic Stimulation (rTMS) [10, 11, 12] and Low‐Intensity Focused Ultrasound Pulsation [13]—assuming a growing role in pDoC treatment.

The inaugural report of pain treatment by stimulating the infraorbital nerve at the infraorbital foramen was documented in a 1967 Science case study [14]. Subsequently, trigeminal nerve stimulation (TNS) has been reported for use in a range of conditions, including craniofacial pain [15, 16, 17, 18, 19], drug‐resistant epilepsy [20, 21], major depressive disorder (MDD) [22, 23, 24, 25], post‐traumatic stress disorder (PTSD) [23, 26], and attention‐deficit/hyperactivity disorder (ADHD) in children [27, 28]. Furthermore, emerging clinical and preclinical studies suggest that TNS may represent a promising neuromodulatory approach for disorders of consciousness (DoC) [29, 30]. By engaging trigeminal afferent pathways and their connections with brainstem arousal systems, autonomic regulatory circuits, and cortical networks, TNS may influence multiple biological processes relevant to consciousness recovery. Nevertheless, the therapeutic application of TNS in DoC remains at an early stage. Its underlying mechanisms, optimal stimulation parameters, appropriate therapeutic targets, long‐term safety profile, and patient selection criteria have not yet been clearly established.

In this narrative review, we summarize the current evidence and conceptual basis for the use of TNS in DoC. We first outline the neuroanatomical and neurophysiological rationale linking trigeminal stimulation to consciousness‐related networks. We then discuss preclinical findings, emerging clinical evidence, and potential mechanisms involving neurotransmitter systems, cerebral hemodynamics, neuroinflammation, and network‐level modulation. Finally, we highlight current limitations and propose future directions for developing mechanism‐driven and clinically standardized TNS strategies for consciousness recovery.

## 2 Literature Search Strategy for This Narrative Review

To support this narrative review, we conducted a targeted literature search of two electronic literature databases (PubMed and Web of Science) for relevant studies on trigeminal nerve stimulation and disorders of consciousness. The search terms included combinations of the following keywords: “TNS”, “trigeminal nerve stimulation”, “Trigeminal nerve electrical stimulation”, “disorders of consciousness”, “consciousness disturbance”, “coma”, “vegetative state”, “unresponsive wakefulness syndrome”, “coma insomnia”, “unconscious”. Studies were considered relevant if they addressed the clinical application of TNS in patients with DoC, preclinical models related to consciousness impairment or brain injury, or mechanistic topics relevant to trigeminal afferent pathways, arousal regulation, cerebral hemodynamics, neuroinflammation, and neuromodulation. Furthermore, the literature had to explicitly describe the surgical procedure and postoperative neuromodulation plan. We also conducted additional searches depend on the references of included articles to identify any other reports related to TNS treatment for DOC.

Because this work is a narrative review, no formal meta‐analysis, risk‐of‐bias assessment, or quantitative evidence grading was performed. Instead, the selected literature was narratively synthesized to summarize the anatomical rationale, preclinical mechanisms, emerging clinical evidence, current limitations, and future perspectives of TNS for DoC. To improve transparency, representative clinical and preclinical studies are summarized in Tables 1 and 2, respectively.

**TABLE 1:** Summary of clinical evidence on TNS for DoC.

Study	Evidence level	Population	Etiology	Main findings	Key limitations	Interpretation
Fan et al. [29] 2019	Single case report/Letter to the Editor	One male; 40	Unconsciousness after pituitary tumor resection	After 4 weeks: spontaneous eye opening and articulated exclamatory speech; GCS increased to 11 After 6 weeks: full awakening; GCS increased to 15 fMRI degree centrality analysis showed brain activity changes: CB_Crus2 ITG IFGtriang PoCG PreCG SMA	Single‐patient observation. Spontaneous recovery could not be excluded Mechanism remains uncertain No long‐term follow‐up outcome reported in the manuscript Outcome assessment mainly relied on GCS, without a more detailed DoC scale such as CRS‐R	Proof‐of‐concept only/Preliminary efficacy signal
Dong et al. [31] 2022	Prospective single‐arm observational case series	13 males, 8 females; 44.29 ± 12.55	11 Traumatic; 7 Stroke; 3 Anoxia	No significant overall CRS‐R improvement at week 4 or follow‐up. 7/9 MCS patients improved, compared with only 1/12 VS/UWS patients recovering to MCS. No major TNS‐related adverse events	Small sample; no control group; short follow‐up period; limited objective biomarkers	Preliminary efficacy signal
Wu et al. [32] 2022	Randomized, double‐blind, placebo‐controlled, parallel‐group clinical study	40 male, 23 female; 52.86 ± 14.26	19 TBI; 12 ABI; 32 CVA	CRS‐R improved after gamma and beta, not sham. Diagnostic improvement: 43.5% in gamma; 25% in beta. Main gains: visual and motor CRS‐R subscales EEG showed frequency‐specific ASSR enhancement CRS‐R change correlated with rhythmic brain activity Responders had better 1‐year GOSE outcomes	Modest sample size. Single‐center study Music and electrical effects are not separable Sham blinding may be imperfect Limited generalizability due to exclusion criteria	Preliminary efficacy signal with mechanistic support; higher‐level evidence but confirmatory trials needed
Ma et al. [30] 2023	Randomized, double‐blind, sham‐controlled clinical trial	25 male, 35 female; 53.6 ± 14.1	10 TBI; 43 Stroke; 7 Anoxia	CRS‐R improved from week 2; GCS from week 4. 50% improved in TNS vs. 22% in sham. FDG‐PET showed increased metabolism in the parahippocampal cortex, precuneus, and MCC. No TNS‐related adverse events	Single‐center design. Modest sample size and short follow‐up. Very small PET/SEP subsamples Long‐term efficacy unclear	Higher‐level evidence but limited by sample size
Wu et al. [33] 2024	Secondary mechanistic EEG connectivity analysis of the previous randomized, double‐blind, placebo‐controlled DoC trial	40 male, 23 female; 52.86 ± 14.26	19 TBI; 12 ABI; 32 CVA	Gamma stimulation increased whole‐scalp gamma‐band PLI The effect was widespread across cortical ROIs Beta stimulation did not increase gamma PLI Sham showed no significant PLI change Gamma PLI change did not correlate with CRS‐R total score change Gamma PLI change correlated with auditory CRS‐R subscore and ASSR changes	Connectivity was estimated at the scalp EEG level, limiting spatial specificity Correlation analyses were not corrected for multiple comparisons Reanalysis of the prior cohort; not an independent efficacy trial	Mechanistic relevance only; not direct therapeutic evidence
Wu et al. [34] 2024	Double‐blind, randomized, crossover mechanistic study in healthy participants	12 males, 42 females; 18–30	/	Acoustic‐electric stimulation improved conscious tactile perception Tactile detection threshold decreased significantly after acoustic‐electric stimulation No clear improvement in auditory perception Acoustic‐sham electric, electric‐only, and sham‐electric conditions showed no systematic benefit Late tactile awareness‐related ERP increased after acoustic‐electric stimulation DCM suggested altered recurrent cortical connections, especially cSI–cSII and iPMC–cSII pathways	Healthy young participants; findings are indirect for DoC therapy Conscious perception task is only a proxy for consciousness The majority of participants were female Music‐specific effects remain unclear Generic MNI coordinates may reduce anatomical precision	Mechanistic relevance only; not direct therapeutic evidence

*Note:* The participants in Study 6 were healthy volunteers.

Abbreviations: ABI, anoxic brain injury; ASSR, auditory steady‐state response; CB_Crus2, cerebellum_Crus2; cSI, contralateral primary somatosensory cortex; cSII, contralateral secondary somatosensory cortex; CVA, cerebrovascular accident; DCM, dynamic‐causal modeling; ERP, event‐related potential; GOSE, Glasgow Outcome Scale‐Extended; IFGtriang, inferior frontal gyrus triangular part; iPMC, ipsilateral premotor cortex; ITG, inferior temporal gyrus; MCC, middle cingulate cortex; MNI, Montreal Neurological Institute; PLI, phase‐lag index; PoCG, postcentral gyrus; PreCG, precentral gyrus; ROIs, regions of interest; SMA, supplementary motor area; TBI, traumatic brain injury.

**TABLE 2:** Preclinical evidence and proposed mechanisms of TNS in brain injury and consciousness‐related models.

Study	Species	Animal model	Electrical stimulation mode						Electrical stimulation target
			Intensity	Freq (Hz)	Pulse width	Duty cycle	Duration	Total	
Zhang et al. [35] 2025	Mice	TBI	0.2 mA	40 Hz	200 microseconds	30 s/min	60 min	once daily for two days	Bilateral V2
Xu et al. [36] 2023	Mice	TBI	0.2 mA	40 Hz	200 microseconds	30 s/min	60 min	/	Bilateral V2
Yang et al. [37] 2022	Male mice	TBI	0.1 mA	10 Hz	200 microseconds	30 s/min	60 min	/	Bilateral V2
Shah et al. [38] 2022	Male rats	SAH	0.5–2.5 V	50 Hz	/	20 s every 5 min	60 min	/	left infraorbital nerve
Zheng et al. [39] 2021	Male rats	TBI‐induced LOC	1 mA	140 Hz	/	10 s on and 5 s off	30 min	/	Bilateral V1
Li et al. [40] 2021	Male rats	TBI + HS	/	50 Hz	/	1 min in every 10 min	60 min	/	Bilateral V1 and V2
Li et al. [41] 2019	Male rats	Hemorrhagic shock	7 V	25 Hz	0.5 ms	1 min in every 4 min	60 min	/	Bilateral V1 and V2
Chiluwal et al. [42] 2017	Male rats	TBI	Direct TNS: 1.0 V; Subcutaneous TNS: 3.0 V	Direct TNS: 25; Subcutaneous TNS: 100 Hz	0.5 ms	1 s on and 1 s off	Direct TNS: 1 min; Subcutaneous TNS: 1 min	Subcutaneous TNS: Total 5 subcutaneous TNS	Bilateral V1

*Note:* HS, hemorrhagic shock; LOC, loss of consciousness; TBI, traumatic brain injury.

## 3 Trigeminal Nerve: Anatomy & Projections

The trigeminal nerve originates within the pons. As the largest cranial nerve, its three branches—the ophthalmic branch (V1), maxillary branch (V2), and mandibular branch (V3)—converge at the trigeminal (Gasserian) ganglion [43, 44, 45]. The nerve connects to the sensory and motor nuclei of the trigeminal nerve, which include the principal sensory nucleus, spinal trigeminal nucleus, mesencephalic nucleus, and motor nucleus. These nuclei project, in part via the ventral trigeminothalamic tract (VTT) and dorsal trigeminothalamic tract (DTT), to the thalamus and the ascending reticular activating system (ARAS) [46]. Through the thalamus and ARAS, they send direct and indirect projections to multiple brain regions, such as the locus coeruleus (LC), nucleus of the solitary tract (NTS), rostral ventrolateral medulla (RVLM), and the cerebral cortex [23, 47] (Figure 1). The unique anatomical features of the trigeminal nerve, which mediate its role as a bridge between external environmental stimulation and functional regulation of the central nervous system (CNS), form the basis for the application of TNS in treating DoC.

**FIGURE 1:**
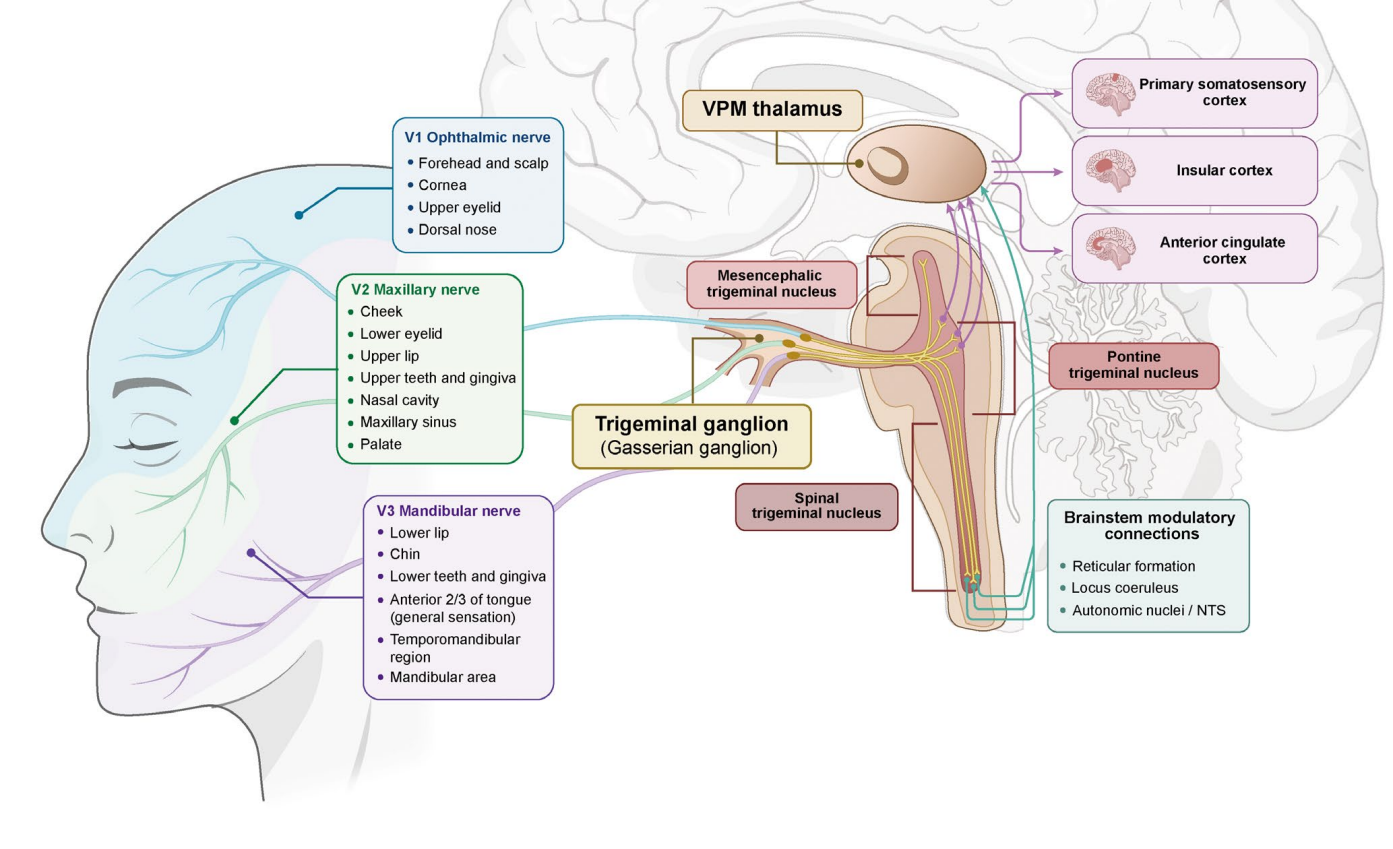
Anatomy and projections of the trigeminal nerve.

## 4 Preclinical Studies

Building upon the unique anatomical structure and connections of the trigeminal nerve, findings from preclinical studies provide a mechanistic rationale for TNS in DoC. Research has confirmed that the hypothalamic neuropeptide hypocretin (also known as orexin‐A, Hcrt) [48] is critical for maintaining wakefulness [49] through its excitatory effects on the cortex [50], hippocampus [51], and other brain regions [51, 52]. In a rat model of traumatic brain injury (TBI), a decrease in orexin levels was observed. TBI‐induced loss of consciousness (LOC) led to the downregulation of EGR1. TNS activates neurons in the spinal trigeminal nucleus (Sp5) and the lateral hypothalamus (LH), which upregulates EGR1 expression in the LH and increases hypocretin (orexin) levels in the cerebrospinal fluid, ultimately leading to an improvement in the level of consciousness [39] (Figure 2a). The aforementioned study also found that TNS improves EEG characteristics by reducing the power of delta waves and increasing the approximate entropy [39]. Since approximate entropy is positively correlated with the level of consciousness [53], these neurophysiological changes ultimately lead to an improvement in behavioral scores in rats. Beyond its known effect of elevating orexin, TNS has also been shown to regulate dopamine secretion in a murine model of TBI. Dopamine (DA), synthesized by mesencephalic neurons of the substantia nigra (SN) and the ventral tegmental area (VTA) [46], is the principal catecholaminergic neurotransmitter in the mammalian brain, governing a broad spectrum of functions—including motor control, cognition, emotion, positive reinforcement, feeding, and neuroendocrine regulation [54]. It has been reported that treatment with low‐dose DA agonists can mitigate cognitive and behavioral dysfunction following severe TBI by increasing DA release and blocking its reuptake [55]. This treatment has also been shown to reduce apoptotic cell death in the hippocampus after severe TBI. The study by Xu et al. [36] proposed that TNS can activate nuclei such as the trigeminal ganglion (TG), paraventricular hypothalamic nucleus (PVN), nucleus of the solitary tract (NTS), parabrachial nucleus (PB), and the LC. Their findings suggest that TNS communicates with the central nervous system via the TG. Their study revealed that the TG is connected to the hippocampus (HPC) through the corticotropin‐releasing hormone (CRH) neurons of the paraventricular hypothalamic nucleus (PVN) and the dopamine transporter (DAT) neurons of the substantia nigra pars compacta/ventral tegmental area (SNc/VTA). Corticotropin‐releasing hormone neurons of the paraventricular hypothalamic nucleus are a critical neuronal subtype for TNS‐induced cognitive enhancement. TNS facilitates dopamine release via the TG → PVN^CRH+^ → SNc^DAT+^/VTA^DAT+^ → HPC neural pathway (Figure 2b). This process reverses TBI‐induced alterations in the hippocampus, including increasing DA levels and modulating gene expression, thereby improving working memory, novel object recognition, and olfactory associative learning in TBI mice. Similarly, Zhang et al. [35] demonstrated that TNS effectively enhances defensive arousal responses in TBI mice by engaging distinct neural circuits mediated by Piezo2 and Tac1 neuronal subpopulations within the trigeminal ganglion. Through the TG^Piezo2+^ → PVN → SNc → DS pathway, TNS facilitates dopamine release in the dorsal striatum (DS), thereby restoring motor function and defensive responses in TBI mice (Figure 2c). These findings suggest that the central dopaminergic system plays a pivotal role in mediating the effects of TNS. The LC is the primary source of noradrenergic innervation to the cortex, representing the other major class of catecholamine (CA) neurons within the cerebral cortex. All LC neurons receive inputs related to autonomic arousal, but distinct subpopulations of those neurons can encode specific cognitive processes, presumably through more specific inputs from forebrain areas [56]. The Locus Coeruleus‐Norepinephrine (LC‐NE) system plays an indispensable role in a variety of autonomic, cognitive, and behavioral functions [57, 58] by virtue of its extensive projections to widespread areas of the brain and spinal cord. These functions include arousal, pupillary control, anxiety generation, attention, decision‐making, and memory [59]. Animal studies have demonstrated that electrical trigeminal nerve stimulation may modulate hippocampal activity via the LC pathway [60]. In the study by Zhang et al. [35], TNS was shown to elevate norepinephrine (NE) levels in the superior colliculus (SC) via the TG^Tac1+^ → LC → SC pathway, thereby restoring visual sensitivity in TBI mice (Figure 2d). These findings collectively suggest a potential role for the LC‐NE system in the mechanism of TNS for treating DoC.

**FIGURE 2:**
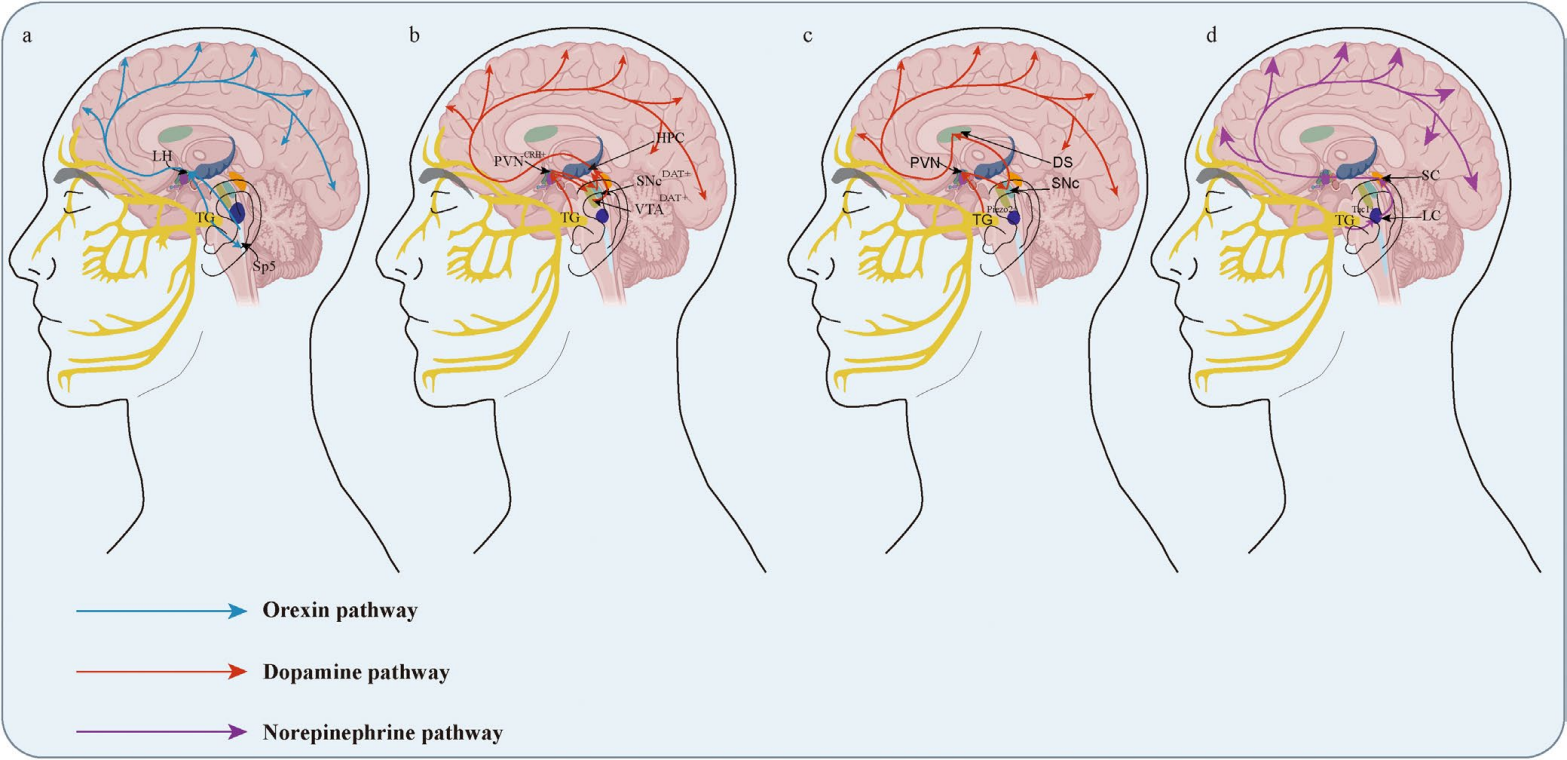
The potential nuclei, neurotransmitters, and pathways involved in TNS for DoC. (a) TNS upregulates cerebrospinal fluid (CSF) hypocretin (orexin) levels via the TG → Sp5 → LH pathway. (b) TNS facilitates dopamine release via the TG → PVN^CRH+^ → SNc^DAT+^/VTA^DAT+^ → HPC pathway. (c) TNS promotes dopamine release via the TG^Piezo2+^ → PVN → SNc → DS pathway. (d) TNS elevates norepinephrine (NE) levels via the TG^Tac1+^ → LC → SC pathway.

In addition to the direct arousal‐promoting effects achieved through the activation of relevant pathways and the release of neurotransmitters such as orexin, dopamine, and norepinephrine, the indirect role of TNS in promoting arousal by modulating cerebral hemodynamics and exerting immunomodulatory effects cannot be overlooked (Figure 3). TNS can activate the antidromic pathway [61], which induces the antidromic release of neurotransmitters such as calcitonin gene‐related peptide (CGRP) [62] and other neuroactive peptides [63], including pituitary adenylate cyclase‐activating polypeptide (PACAP), nitric oxide (NO), substance P, adenosine triphosphate (ATP), and neurokinin A. This process subsequently promotes vasodilation and increases cerebral blood flow [63, 64]. Due to the convergence of the parasympathetic branches of the facial nerve with the trigeminal nerve at the sphenopalatine ganglion (SPG) [65], TNS can activate the trigeminal‐parasympathetic pathway. This activation leads to parasympathetic vasodilation within the cerebral vasculature [66, 67], which is likely mediated by the release of neurotransmitters such as acetylcholine (ACh) [68, 69] and vasoactive intestinal peptide (VIP) [70] from the parasympathetic system. The trigeminal ganglion sends direct projections to the rostral ventrolateral medulla (RVLM), which is a critical medullary nucleus responsible for systemic vasomotor control [71, 72]. The central pathway, which arises from the brainstem projections of the trigeminal ganglion, activates a population of cells in the RVLM upon trigeminal nerve stimulation; this activation causes peripheral vasoconstriction via adrenergic signaling, subsequently increasing the mean arterial pressure (MAP) [73]. Concurrently, the increase in cerebral blood flow (CBF) induced by RVLM activation via its intrinsic projections is mediated by NO [71, 74]. Thus, TNS improves hemodynamics by increasing MAP, CBF, and brain tissue oxygen tension (PbrO_2_), while decreasing cerebrovascular resistance (CVR) [42], This is accompanied by a reduction in peri‐lesional volume [40, 42]. In traumatic models, TNS increases cerebrospinal fluid CGRP concentration and downregulates the cortical expression of intercellular adhesion molecule‐1 (ICAM‐1), a specific early marker of vasospasm [75]. These changes result in an increase in the luminal diameter of the internal carotid artery (ICA), middle cerebral artery (MCA), and anterior cerebral artery (ACA), prevent vasoconstriction in cortical arterioles, mitigate subarachnoid hemorrhage (SAH)‐induced cerebral vasospasm (CV), and reduce the formation of cerebral microthrombi [38]. Furthermore, TNS ameliorates TBI‐induced blood–brain barrier (BBB) disruption [42] and mitigates cerebral edema [37, 42] by upregulating the expression of the tight junction protein claudin‐5 [37]. It also reduces levels of the pro‐inflammatory cytokines tumor necrosis factor α (TNF‐α) and interleukin‐6 (IL‐6) [37, 40, 41, 42] and suppresses cellular apoptosis [37, 38]. These integrated effects ultimately ameliorate neurological deficits in animal models, leading to improved performance on the Neurological Severity Score (NSS) and Wire Grip and Motion Test (WGMT) [37]. By modulating autonomic nervous system (ANS) activity in hemorrhagic shock (HS) models, TNS alleviates severe HS‐induced sympathoexcitation and stimulates norepinephrine release. This action prevents hypovolemia‐induced reductions in mean arterial and cerebral perfusion pressures, conferring protection against hypoxic–ischemic neurological injury [41].

**FIGURE 3:**
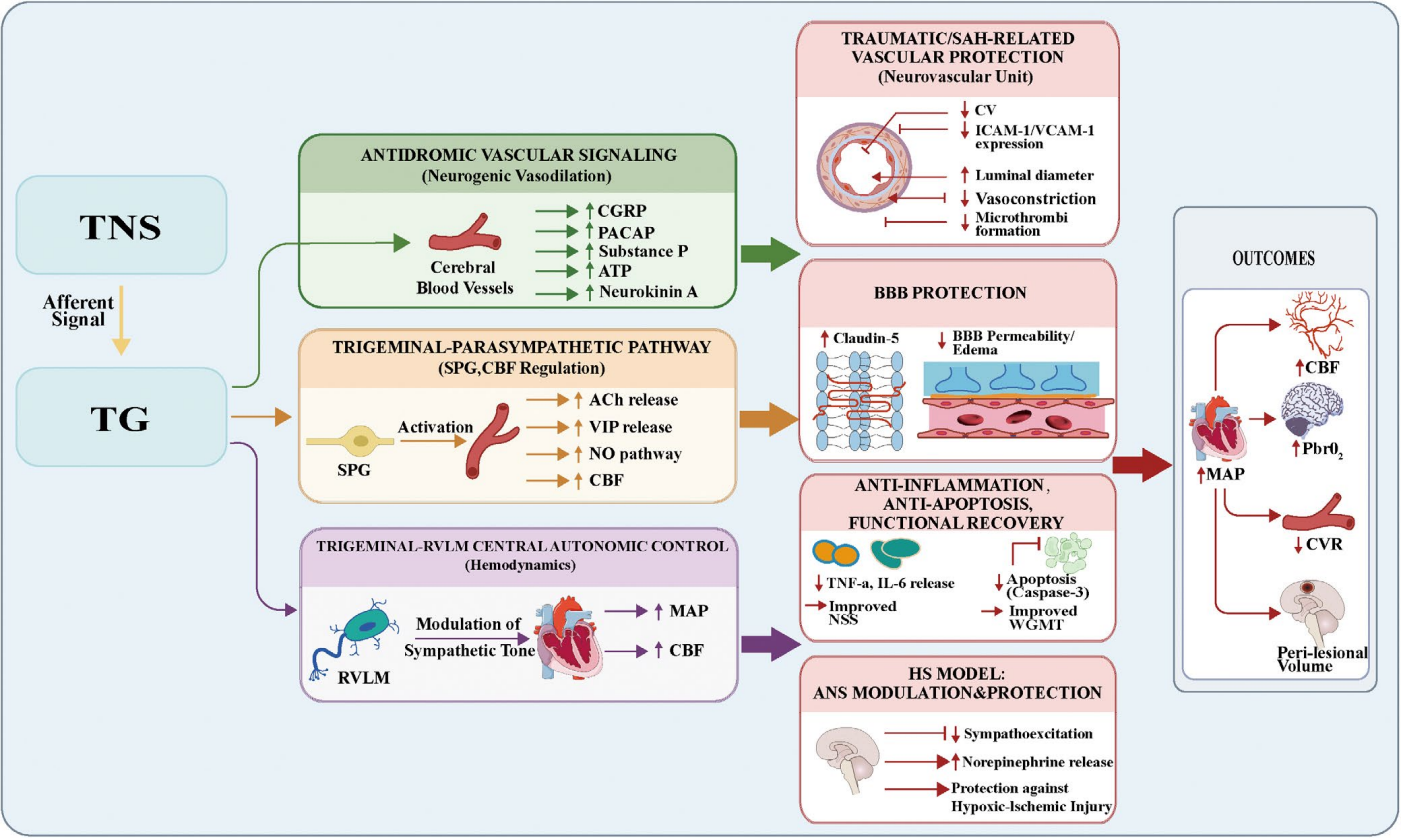
Schematic representation of the multimodal mechanisms underlying TNS: from hemodynamic regulation to neuroprotection. Solid arrows denote activation or increase; T‐bars denote inhibition or reduction. CGRP, calcitonin gene‐related peptide; CVR, cerebrovascular resistance; ICAM‐1, intercellular adhesion molecule‐1; NSS, Neurological Severity Score; PACAP, pituitary adenylate cyclase‐activating polypeptide; PbrO_2_, brain tissue oxygen tension; RVLM, rostral ventrolateral medulla; SPG, sphenopalatine ganglion; TG, trigeminal ganglion; TNS, trigeminal nerve stimulation; WGMT, Wire Grip and Motion Test.

## 5 Clinical Evidence

Current literature has reported clinical trials investigating TNS for the treatment of DoC. The 2019 case report by Fan et al. is the earliest accessible published record to date [29]. It documents a 40‐year‐old male who developed DoC following pituitary tumor resection, which was complicated by secondary lateral ventriculomegaly. Despite receiving multiple resuscitation measures, including hyperbaric oxygen therapy, comprehensive sensory stimulation, median nerve stimulation, and pharmacotherapy, his condition showed no improvement. However, the V1 and V2 were stimulated by placing two pairs of electrode pads over the bilateral supraorbital foramina and infraorbital foramina. Following 4 weeks of TNS treatment, the patient began to demonstrate spontaneous eye opening and exclamatory articulated speech, with an improved Glasgow Coma Scale (GCS) score. After 6 weeks of TNS, the patient became fully alert (GCS = 15). A comparison of the patient's fMRI images before and after treatment revealed modulated cerebral blood flow and metabolism. As the first documented case of successful coma reversal using TNS, this case report provides early proof‐of‐concept observations suggesting that TNS may be feasible and clinically relevant in selected patients. These findings offer promising preliminary evidence to support future clinical trials investigating TNS for the treatment of disorders of consciousness. However, due to the absence of a control group, the possibility of spontaneous recovery, concurrent rehabilitation interventions, and individual differences in injury severity and recovery trajectories, causal therapeutic efficacy cannot be established.

Dong et al. reported a prospective single‐arm observational case series involving 21 chronic DoC patients who received non‐invasive trigeminal nerve stimulation [31]. In their study, although the overall CRS‐R scores did not significantly improve after treatment, behavioral improvements were observed in a subset of patients, particularly those diagnosed with MCS. These findings may suggest a preliminary efficacy signal and indicate that TNS is feasible and generally well tolerated in the short term. As a small observational study, their findings suggest that the therapeutic effects of TNS may differ across different subgroups of patients with DoC. The lack of significant improvement in overall CRS‐R scores after treatment in their findings may be attributable to several factors, including the small sample size, the absence of a sham/control group, the short follow‐up duration, the still uncertain therapeutic efficacy, and the limited availability of objective biomarkers.

Subsequently, a randomized, double‐blind, sham‐controlled trial [30] provided evidence suggesting that patients in the TNS treatment group tended to exhibit greater improvements in the Coma Recovery Scale‐Revised (CRS‐R) and Glasgow Coma Scale (GCS) scores compared to the sham‐control group. Furthermore, the number of patients who transitioned to the MCS or emerged from MCS was notably higher in the TNS group, suggesting a potential clinical benefit of TNS in promoting recovery of consciousness. Subgroup analyses pointed toward superior treatment effects that may be more pronounced in patients with VS, shorter disease duration, and those whose disorders of consciousness were caused by TBI or stroke. Fluorodeoxyglucose positron emission tomography (FDG‐PET) imaging indicated increased cerebral metabolism in the parahippocampal cortex, precuneus, and mid‐cingulate cortex. Beyond unimodal electrical stimulation, multimodal stimulation may offer additional therapeutic value for DoC. The potential benefit of this approach may lie in its ability to engage more extensive sensory afferent networks and circuits, thereby possibly promoting a more widespread and orchestrated pattern of brain activation [76, 77]. Ma et al. provide higher‐level clinical evidence from a randomized double‐blind sham‐controlled trial, suggesting that non‐invasive TNS may improve behavioral recovery and local brain metabolism in pDoC; however, the findings remain limited by a single‐center design, a modest sample size, a short follow‐up, and small PET/SEP subsamples.

In a double‐blind, placebo‐controlled, parallel‐group study [32], acoustic‐electric TNS was reported to enhance consciousness and to modulate brain oscillations at the stimulation frequency. Post‐treatment, diagnostic improvements were observed in 43.5% of patients in the gamma‐frequency group and 25% in the beta‐frequency group, suggesting a possible frequency‐dependent therapeutic effect. These therapeutic effects appeared to persist at the 1‐year follow‐up, and the observed improvement in consciousness appeared to be functionally correlated with an increase in rhythmic brain activity. Subsequent research found that gamma acoustic‐electric TNS enhanced resting‐state functional connectivity in the gamma band across widespread cortical regions, but not in the beta band [33]. The absence of this enhancement after beta‐frequency or sham stimulation supports the possibility of frequency specificity. Critically, increases in the gamma‐band phase‐lag index were selectively associated with gains in the auditory steady‐state response and the CRS‐R auditory subscale, whereas no comparable association was observed with the total CRS‐R score, pointing toward a specific mechanism within the auditory processing domain. The above findings suggest that the enhancement of gamma‐band connectivity by gamma acoustic‐electric TNS may not be fully explained by spontaneous recovery, placebo effects, or general sensory stimulation. They provided randomized, double‐blind, placebo‐controlled evidence that rhythmic musical‐electrical TNS may improve consciousness in DoC patients, especially through frequency‐specific enhancement of oscillatory brain activity, but the findings remain preliminary because of sample size, sham‐blinding, and modality‐disentanglement limitations.

The mechanistic rationale is partly supported by evidence from healthy participants showing that acoustic‐electric TNS [34], but not acoustic‐sham or electric‐only stimulation, was associated with improvements in conscious tactile perception and increased late awareness‐related ERP responses. Dynamic causal modeling indicated that these effects may be related to changes in inter‐regional cortical connectivity within recurrent somatosensory–premotor networks, suggesting that multimodal TNS could potentially facilitate consciousness‐related processing by modulating cortical recurrent processing rather than by peripheral stimulation alone. Nevertheless, current evidence remains limited by several issues: the therapeutic efficacy for DoC has not yet been directly established; conscious perception tasks serve only as a proxy for consciousness; the majority of participants are female; the role of concomitant music stimulation remains unclear; and the use of generic MNI coordinates may compromise anatomical precision. Collectively, these studies suggest a possible advantage of multimodal stimulation in TNS therapy, although further direct evidence in DoC populations is needed.

Taken together, the available clinical evidence suggests that TNS is a promising yet still investigational neuromodulatory approach for DoC, with the strongest clinical support derived from the sham‐controlled trial by Ma et al. and the controlled acoustic–electric stimulation study by Wu et al. Subgroup evidence remains preliminary (Table 3): Dong et al. suggested better responses in MCS, whereas Ma et al. reported greater CRS‐R improvement in VS but with very few MCS patients. From an etiological perspective, Ma et al. have demonstrated a stronger signal in traumatic brain injury (TBI) and stroke than in anoxia, whereas the patient‐level pattern reported by Dong et al. is broadly concordant, though it has not been subjected to statistical testing.

**TABLE 3:** Subgroup evidence of clinical evidence on TNS for DoC.

Study	Etiology	DoC subtype subgroup	Subgroup findings	Interpretation
Fan et al. [29] 2019	Unconsciousness after pituitary tumor resection	Single PVS case	The patient improved from severe unconsciousness to full awakening after 6 weeks of TNS	Cannot infer subtype‐ or etiology‐specific efficacy; useful only as initial feasibility evidence
Dong et al. [31] 2022	11 Traumatic; 7 Stroke; 3 Anoxia	12 VS/UWS; 9 MCS	MCS appeared more responsive: 7/9 MCS improved, whereas only 1/12 VS/UWS improved or recovered to MCS. Descriptive inference from individual patient data: most responders were traumatic cases; stroke response was limited; anoxic cases showed no clear improvement in their Table	Suggests a stronger preliminary signal in MCS than VS/UWS, but uncontrolled design limits inference
Wu et al. [32] 2022	19 TBI; 12 ABI; 32 CVA	42 MCS; 21 UWS	Overall diagnostic improvement occurred in 43.5% of the gamma group and 25% of the beta group; formal subtype‐specific and etiology‐specific treatment effects were not reported	Provides controlled evidence for overall efficacy but limited for subgroup‐based clinical selection
Ma et al. [30] 2023	10 TBI; 43 Stroke; 7 Anoxia	TNS group: 27 VS, 3 MCS; sham group: 26 VS, 4 MCS	CRS‐R improvement was statistically higher in VS than MCS; TBI and stroke benefited more than anoxia	Suggests possible benefit in VS, but interpretation is limited by very small MCS numbers; anoxia causes more extensive cortical damage than TBI/stroke → lower responsiveness to peripheral wake‐promoting stimuli
Wu et al. [33] 2024	19 TBI; 12 ABI; 32 CVA	42 MCS; 21 UWS	Gamma acoustic‐electric TNS increased gamma‐band functional connectivity; beta and sham did not; no subtype/etiology subgroup analysis reported	Supports a gamma‐specific mechanism but not subgroup‐specific therapeutic prediction
Wu et al. [34] 2024	Healthy participants only	Healthy participants only	No DoC subtype or etiology subgroup analysis	Supports mechanistic plausibility of multimodal acoustic‐electric TNS, not direct DoC subgroup efficacy

## 6 Translational Challenges and Future Research Roadmap

Although TNS has increasingly been investigated as a potentially promising neuromodulatory strategy for DoC, its clinical translation may remain limited by substantial heterogeneity in stimulation targets, stimulation parameters, patient populations, mechanistic interpretation, and preclinical models. Current evidence appears to support the feasibility and short‐term safety of non‐invasive TNS, but it remains insufficient to confidently define an optimized therapeutic protocol or to reliably identify the DoC subgroups most likely to benefit. Therefore, future research should aim to move beyond proof‐of‐concept observations and gradually establish a staged translational roadmap integrating parameter optimization, long‐term clinical evaluation, mechanism validation, and disease‐relevant preclinical model development.

### 6.1 Parameter Optimization and Standardization of Stimulation Protocols

Variations in the choice of stimulation modalities, targets, and parameters could shape the therapeutic mechanisms, efficacy, and safety of TNS in treating DoC. For instance, the spatial divergence in the anatomical locations of the V1, V2, and V3 branches relative to the trigeminal ganglion may lead to differential neuromodulatory effects at the stimulation target, resulting in variability in the consistency of clinical outcomes. Based on the mode of stimulation, clinically available TNS can be classified into two main approaches: non‐invasive eTNS delivered transcutaneously via skin patch electrodes and invasive electrical stimulation. Invasive electrode implantation is further subdivided into electrical stimulation of the peripheral nerve branches (V1, V2, V3), known as trigeminal peripheral neurostimulation (PNS) [78, 79] and Gasserian ganglion stimulation (GGS) [15, 16, 17]. No consensus has been reached regarding the optimal stimulation parameters for different stimulation modalities and targets. Invasive electrode stimulation allows for direct targeting of nerves or ganglia, requiring lower stimulation intensity and producing more pronounced effects. Although it involves less trauma and risk compared to other neurosurgical procedures, it carries greater trauma and risk relative to eTNS. Furthermore, the implantation of invasive electrodes must be performed by an experienced neurosurgeon. The eTNS offers advantages such as non‐invasiveness, a favorable safety profile, ease of clinical application, and the use of disposable electrodes. However, as it does not act directly on the nerve, it often requires higher stimulation parameters, and its effects can be attenuated by the skull and scalp tissues. Owing to considerations of safety, ease of operation, and cost‐effectiveness, current clinical studies predominantly adopt eTNS [29, 30, 31, 32, 33, 34]. In contrast, preclinical studies more often choose invasive electrode stimulation [37, 38, 39, 40, 41, 42] over eTNS [35, 36], possibly because of the limited surface area available on mice or rats for affixing electrode patches. Nevertheless, it remains uncertain whether invasive electrical stimulation and eTNS produce fully equivalent mechanisms and effects.

Across available studies, trigeminal nerve stimulation for disorders of consciousness shows substantial heterogeneity in both peripheral targets and stimulation parameters (Table 4). TNS protocols mainly target the ophthalmic and maxillary branches of the trigeminal nerve, usually through electrodes placed around the supraorbital/superior orbital and infraorbital regions. This approach was intended to provide peripheral trigeminal afferent input to brainstem, thalamic, and cortical arousal networks. In contrast, acoustic‐electric TNS protocols targeted the maxillary and mandibular branches through bilateral middle and lower facial electrodes, while synchronizing electrical stimulation with rhythmic musical stimulation. This represents a shift from purely peripheral arousal stimulation toward frequency‐specific modulation of thalamocortical oscillations. Parameter settings also vary considerably. Although 40 Hz is the most consistently used frequency across studies, its interpretation differs: in eTNS it functions as a standard stimulation frequency, whereas in acoustic‐electric TNS it is explicitly designed to entrain gamma‐band neural activity. Current intensity ranges from 18 to 20 mA in early electrical protocols, to 10–15 mA in the randomized sham‐controlled trial, and to ±8 mA in the acoustic‐electric patient studies. Treatment dose ranges from several hours per day over 4–6 weeks in conventional TNS studies to 40 min per day over five consecutive days in rhythmic acoustic‐electric paradigms. Overall, these differences indicate that the field has not yet established an optimal target or dose–response profile. Existing evidence supports the feasibility of non‐invasive TNS, but future trials should directly compare trigeminal targets, stimulation intensity, frequency, session duration, and treatment course to define the most effective protocol for specific DoC subgroups.

**TABLE 4:** Comparison of stimulation modalities, targets, and parameters.

Study	Population	Stimulation modality	Stimulation targets	Parameters
Fan et al. [29] 2019	One male; 40	eTNS	Bilateral ophthalmic nerve V1 and maxillary nerve V2	18–20 mA; 40 Hz; pulse width 200 μs; 30 s/min; 6 h/day; 6 weeks
Dong et al. [31] 2022	13 male, 8 female; 44.29 ± 12.55	eTNS and conventional rehabilitation	Bilateral ophthalmic nerve V1 and maxillary nerve V2	18–20 mA; 40 Hz; pulse width reported as 200 ms; 30 s/min; 3 h/session, twice daily; 4 weeks
Wu et al. [32] 2022	40 male, 23 female; 52.86 ± 14.26	Acoustic‐electric eTNS	Electrical stimulation of V2/V3 and synchronized musical stimulation	Acoustic stream 40 min/day for 5 days; gamma 40 Hz, beta 28 Hz, or sham; music at 50 dB SPL; electrical sinusoidal stimulation via bilateral middle/lower facial electrodes
Ma et al. [30] 2023	25 male, 35 female; 53.6 ± 14.1	eTNS and conventional rehabilitation	Bilateral ophthalmic nerve V1 and maxillary nerve V2	10–15 mA; 40 Hz; pulse width 200 μs; 30 s on/30 s off; 3 h/day; 5 days/week; 4 weeks; sham current 0 mA
Wu et al. [33] 2024	40 male, 23 female; 52.86 ± 14.26	Acoustic‐electric eTNS	Electrical stimulation of V2/V3 and synchronized musical stimulation	Acoustic stream 40 min/day for 5 days; gamma 40 Hz, beta 28 Hz, or sham; music at 50 dB SPL; electrical sinusoidal stimulation via bilateral middle/lower facial electrodes
Wu et al. [34] 2024	12 males, 42 females; 18–30	Acoustic‐electric eTNS	Electrical stimulation of V2/V3 and synchronized musical stimulation	20 min; 40 Hz; ±4 mA; 3 × 3 cm electrodes; impedance < 10 kΩ; music at 70.8 dB SPL

Abbreviation: SPL, sound pressure levels.

Future trials should therefore be designed to define a reproducible dose–response relationship rather than simply comparing active stimulation with sham stimulation. A first step would be to establish a standardized reporting framework including stimulation modality, electrode location, trigeminal branch targeted, waveform, current intensity, pulse width, frequency, duty cycle, impedance, session duration, total treatment duration, and adverse events. Subsequent studies should use controlled dose‐finding designs to compare different stimulation intensities, frequencies, treatment durations, and anatomical targets within the same study population. Factorial or adaptive trial designs may be particularly useful because they allow systematic evaluation of multiple stimulation variables while reducing sample‐size requirements. Stratification by DoC subtype, etiology, disease duration, baseline CRS‐R profile, age, and comorbidities should also be incorporated, as patients in unresponsive wakefulness syndrome/vegetative state and minimally conscious state may respond differently to TNS. This strategy is necessary to disentangle the unique contributions of stimulation parameters and sites to the mechanisms, safety, and efficacy of TNS in DoC. Our findings will provide the necessary evidence for establishing standardized protocols, enabling clinicians to select the most effective stimulation sites and parameters.

### 6.2 Long‐Term Safety, Durability, and Clinical Effectiveness

Current clinical evidence has not reported serious adverse events, but long‐term safety remains insufficiently characterized. This is particularly important because DoC patients may require repeated or prolonged stimulation, and many are medically fragile, elderly, pediatric, or dependent on implanted medical devices such as pacemakers, spinal cord stimulators, or deep brain stimulation systems. Future studies should therefore include extended follow‐up periods rather than relying only on short‐term post‐treatment assessments.

Long‐term safety monitoring should include local skin reactions, pain, headache, trigeminal sensory disturbance, autonomic instability, cardiovascular effects, sleep–wake disruption, seizure risk, and potential interference with implanted devices. In addition, repeated assessment of vital signs, electroencephalography, autonomic markers such as heart‐rate variability, and neuroimaging safety markers may help determine whether prolonged TNS produces sustained or maladaptive central nervous system effects.

Long‐term effectiveness should also be evaluated more rigorously. Future randomized controlled trials should include repeated CRS‐R assessments at baseline, during treatment, immediately after treatment, and at delayed follow‐up time points such as 3, 6, and 12 months. Outcomes should extend beyond total CRS‐R score and include diagnostic transition, command‐following, auditory and visual function, sleep–wake organization, functional outcome scales, caregiver burden, and durability of response after stimulation discontinuation. Because spontaneous recovery remains a major confounder in DoC research, future trials should include sham stimulation, blinded assessment, repeated baseline measurements, and careful adjustment for time since injury and etiology.

### 6.3 Mechanism Validation and Biomarker‐Guided Translation

By reviewing previous preclinical and clinical findings, we summarized the currently proposed mechanisms underlying TNS for the treatment of DoC (Table 5). Among these, clinical studies suggest that TNS may modulate thalamocortical oscillations, enhance gamma‐band functional connectivity, and reactivate metabolism in consciousness‐related cortical and limbic regions. However, these mechanisms still require further validation. In addition, among the clinical studies we reviewed, some reported no significant improvement in Coma Recovery Scale–Revised (CRS‐R) scores in patients with DoC after TNS treatment [31]. Moreover, one study suggested that patients in a vegetative state may respond better to TNS [30], whereas other studies indicated that patients in MCS may benefit more than those in VS [31, 32]. These inconsistent clinical findings are clearly unsatisfactory and may be attributable to mechanisms that have not yet been fully elucidated.

**TABLE 5:** Proposed mechanisms of TNS in DoC and level of supporting evidence.

Evidence source	Proposed mechanism	Putative pathway	Main supporting findings	Level of supporting evidence	Interpretation
Case report; Fan et al. [29] 2019	Clinical arousal signal from early case evidence	Peripheral trigeminal input may activate brainstem reticular/arousal systems and modulate brain connectivity or metabolism	A case report described awakening after continuous TNS, but spontaneous recovery could not be excluded	Low‐level clinical support	Proof‐of‐concept only; useful for historical rationale but insufficient to infer efficacy or mechanism
DoC clinical trial; Wu et al. [32] 2022	Restoration of thalamocortical oscillations	Rhythmic acoustic‐electric TNS entrains gamma/beta oscillatory activity in thalamocortical networks	Musical‐electrical TNS improved consciousness diagnosis in a subset of DoC patients; behavioral benefit correlated with stronger oscillatory responses at the stimulation frequency	Direct clinical support	Evidence‐supported mechanism; likely relevant to network‐level consciousness recovery
DoC EEG connectivity study; Wu et al. [33] 2024	Enhancement of gamma‐band functional connectivity	Increased synchronization across distributed cortical networks, especially gamma‐band PLI	Gamma acoustic‐electric TNS increased gamma‐band functional connectivity; the effect was frequency‐specific and absent after beta or sham stimulation	Direct clinical support	Evidence‐supported mechanism; supports restoration of large‐scale cortical communication
Randomized sham‐controlled pDoC study; Ma et al. [30] 2023	Metabolic reactivation of consciousness‐related regions	Increased activity in the precuneus, cingulate, parahippocampal, and limbic–cortical network nodes	TNS improved CRS‐R/GCS scores and increased FDG‐PET metabolism in the right parahippocampal cortex, right precuneus, and bilateral middle cingulate cortex	Direct clinical support	Evidence‐supported but spatially limited; the mechanism requires replication with larger imaging cohorts
Healthy‐human mechanistic study; Wu et al. [34] 2024	Improvement of sensory processing and conscious perception	Enhanced sensory gain and recurrent cortical processing after multimodal stimulation	Acoustic‐electric stimulation improved tactile conscious perception in healthy participants and was associated with changes in inter‐regional cortical connectivity	Indirect clinical support	Mechanistic relevance only; supports plausibility but not direct therapeutic evidence in DoC
Preclinical LOC/TBI model; Zheng et al. [39] 2021	Activation of brainstem–hypothalamic arousal circuits	TG → Sp5 → LH → hypocretin/orexin signaling	TNS activated Sp5 and LH neurons, improved EEG and behavioral consciousness measures, and upregulated hypocretin in TBI‐induced LOC rats	Preclinical support	Hypothesis‐generating; plausible arousal pathway requiring validation in patients
Preclinical TBI circuit study; Zhang et al. [35] 2025	Noradrenergic arousal modulation	TG^Piezo2+^ → LC → SC → NE release	TNS increased noradrenaline in the SC and improved defensive arousal responses after TBI	Preclinical support	Hypothesis‐generating; may explain improved sensory responsiveness and vigilance
Preclinical TBI studies; Xu et al. [36] 2023; Zhang et al. [35] 2025	Dopaminergic mesocircuit modulation	TG → PVN → SNc/VTA → HPC or DS → dopamine release	TNS increased hippocampal dopamine through TG–PVN–SNc/VTA–HPC circuitry and improved cognition; TGPiezo2 + –PVN–SNc–DS pathway promoted dopamine release and motor recovery	Preclinical support	Hypothesis‐generating; relevant to dopaminergic mesocircuit models of DoC
Preclinical severe TBI model; Chiluwal et al. [42] 2017	Cerebral perfusion and oxygenation enhancement	RVLM‐mediated pressor response; trigemino‐cerebrovascular vasodilation; trigemino‐parasympathetic reflex	TNS increased systemic blood pressure, CBF, and PbrO2 in severe TBI rats	Preclinical support	Plausible especially in acute/subacute injury; direct evidence in chronic DoC is lacking
Preclinical hemorrhagic shock model; Li et al. [41] 2019	Autonomic regulation and endogenous resuscitative response	Sympathetic–parasympathetic coactivation, norepinephrine release, endogenous pressor response, and improved brain perfusion	In severe hemorrhagic shock rats without fluid resuscitation, TNS improved 60‐min survival, delayed hemodynamic decompensation, increased norepinephrine, improved brain perfusion, induced low‐frequency systemic blood‐pressure oscillations, and reduced systemic inflammation	Preclinical support	Hypothesis‐generating; may be relevant to acute brain injury, hypoperfusion, or autonomic instability, but not directly validated in DoC
Preclinical TBI + HS model; Li et al. [40] 2021	Low‐frequency CBF oscillation and penumbral protection	TNS‐induced CBF oscillations improve oxygen delivery and endothelial function	TNS‐induced CBF oscillations reduced lesion volume, hypoxic injury, neuroinflammation, increased eNOS, improved neurological recovery and survival in TBI plus hemorrhagic shock	Preclinical support	Hypothesis‐generating; likely more relevant to acute hypoperfusion than chronic DoC
Preclinical SAH model; Shah et al. [38] 2022	Microcirculatory protection and CGRP‐mediated vasodilation	Trigeminal vascular afferents release vasoactive mediators, improve macro‐ and microcirculation	TNS reduced vasospasm, microthrombosis, ICAM‐1, hypoxic injury, and apoptosis, while increasing CSF CGRP in SAH rats	Preclinical support	Hypothesis‐generating; supports the vascular‐neuroprotective rationale
Preclinical TBI models; Chiluwal et al. [42] 2017; Yang et al. [37] 2022	BBB protection, anti‐inflammatory, and anti‐apoptotic effects	Reduced secondary injury cascade after brain trauma	TNS reduced edema, BBB disruption, lesion volume, TNF‐α, IL‐6, and apoptosis‐related markers in TBI models	Preclinical support	Hypothesis‐generating; may support neuroprotection but not yet proven as a DoC recovery mechanism

Furthermore, in preclinical studies, many proposed pathways, including brainstem‐hypothalamic arousal circuits, locus coeruleus‐noradrenergic signaling, dopaminergic mesocircuit modulation, cerebral perfusion enhancement, anti‐inflammatory effects, blood–brain barrier protection, and microcirculatory improvement, are mainly derived from preclinical models. These mechanisms are biologically plausible, but their direct relevance to human DoC has not yet been fully demonstrated. And dynamic long‐term tracking of neuroplastic changes and synaptic remodeling after TNS intervention remains limited in preclinical studies.

Future studies should distinguish clearly between evidence‐supported mechanisms and hypothesis‐generating mechanisms. To validate candidate mechanisms in patients, TNS trials should incorporate multimodal biomarkers, including EEG spectral power, functional connectivity, event‐related potentials, FDG‐PET metabolism, fMRI connectivity, fNIRS hemodynamics, autonomic indices, pupillometry, and circulating inflammatory or neurotrophic markers. These biomarkers should be measured longitudinally to determine whether neural changes precede, accompany, or follow behavioral recovery.

Mechanism validation should also be causal rather than purely correlational. In preclinical studies, circuit‐specific tools such as optogenetics, chemogenetics, neurochemical sensors, pathway tracing, and selective lesion or blockade approaches could be used to test whether specific trigeminal‐brainstem‐thalamocortical, trigeminal‐hypothalamic, noradrenergic, or dopaminergic circuits are necessary for TNS‐induced arousal and behavioral improvement. In clinical studies, biomarker‐guided stratification may help identify responders and non‐responders. For example, patients with preserved thalamocortical connectivity, residual auditory processing, or measurable gamma‐band entrainment may be more likely to benefit from specific stimulation paradigms.

### 6.4 Development of Disease‐Relevant and Translationally Valid Preclinical Models

The lack of appropriate preclinical models is another major obstacle. Most existing animal studies use acute models such as TBI, SAH, or HS. These models are valuable for studying acute neuroprotection, cerebral perfusion, and arousal responses, but they do not fully reproduce the chronic, heterogeneous, and multi‐etiological nature of human DoC. Moreover, species differences in trigeminal anatomy, skull structure, nerve depth, electrode placement, and current distribution may limit direct translation from rodents to humans.

Future preclinical work should establish a standardized model system that reflects the major clinical etiologies of DoC, including traumatic brain injury, hypoxic–ischemic injury after cardiac arrest, ischemia–reperfusion injury, hemorrhagic brain injury, toxic‐metabolic injury, and mixed injury mechanisms. Models should also incorporate clinically relevant variables such as age, sex, comorbid cardiovascular disease, autonomic dysfunction, and prolonged post‐injury time windows. Chronic models are especially needed to determine whether TNS can promote sustained recovery rather than only transient arousal or acute neuroprotection.

To improve translational validity, animal studies should include anatomically precise stimulation mapping, computational modeling of electric‐field distribution, and parameter scaling across species. Outcome measures should combine behavioral arousal assessment, sleep–wake architecture, EEG activity, sensory responsiveness, neuroimaging, neurochemical measurements, inflammatory profiling, and histological analysis. Larger animal models may also be useful for validating electrode placement, stimulation tolerability, and device‐related safety before complex clinical translation.

### 6.5 Proposed Future Research Roadmap

In summary, the future development of TNS for DoC may benefit from proceeding through a structured translational pathway (Table 6). First, stimulation protocols would ideally be standardized and optimized through harmonized reporting, dose‐finding studies, and direct comparison of stimulation targets and parameters across clinically relevant DoC subgroups. Second, long‐term randomized trials would be valuable to assess safety, durability, and clinical effectiveness. Third, multimodal biomarkers and causal preclinical experiments could be employed to further probe the mechanisms underlying TNS‐induced recovery. Finally, disease‐relevant and anatomically informed preclinical models could be developed not only to help bridge the gap between experimental neurostimulation and clinical application, but also to support biomarker‐guided and response‐adaptive TNS strategies for potentially more individualized treatment of DoC patients.

**TABLE 6:** Proposed future research roadmap.

Stage	Core objective	Key research questions	Recommended study design	Expected translational output
Stage 1: Protocol standardization	Harmonize reporting standards and define target‐ and dose–response relationships	Which stimulation modality, trigeminal branch, electrode location, intensity, frequency, pulse width, duty cycle, session duration, and treatment course are most appropriate for different DoC subgroups?	Consensus reporting framework; harmonized protocol registry; sham‐controlled dose‐finding trials; factorial or adaptive designs; subgroup stratification by diagnosis, etiology, disease duration, and baseline neural biomarkers	Standardized reporting criteria and candidate optimized TNS protocols for VS/UWS, MCS, and etiology‐specific DoC populations
Stage 2: Long‐term clinical validation	Assess safety, durability, and real‐world effectiveness	Are treatment effects sustained, and what are the long‐term risks?	Multicenter randomized controlled trials with 3‐, 6‐, and 12‐month follow‐up	Evidence for long‐term safety, efficacy, and clinical applicability
Stage 3: Mechanism validation	Identify causal neural and systemic pathways	Does TNS restore consciousness through thalamocortical, brainstem, autonomic, vascular, inflammatory, or dopaminergic mechanisms?	Multimodal EEG/PET/fMRI/fNIRS studies; autonomic and molecular biomarkers; causal preclinical circuit studies	Biomarkers of response and mechanistic targets for protocol refinement
Stage 4: Translational model development and precision neuromodulation	Build disease‐relevant preclinical models and use them to inform individualized TNS strategies	Which models best reproduce human DoC etiologies, chronicity, age, comorbidities, and stimulation‐target anatomy? Can baseline biomarkers and early neural responses guide individualized stimulation protocols?	Chronic multi‐etiology animal models; species‐specific anatomical mapping; computational electric‐field modeling; multimodal behavioral, electrophysiological, imaging, autonomic, and molecular readouts; biomarker‐guided and response‐adaptive stimulation paradigms	Reproducible preclinical platforms for mechanism and safety testing, together with biomarker‐informed strategies for personalized TNS treatment in selected DoC patients

## 7 Conclusion

Leveraging the unique anatomy of the trigeminal nerve, TNS has been hypothesized to potentially promote recovery of consciousness in preclinical models by engaging specific pathways, modulating the release of key neurotransmitters (hypocretin, DA, NE), and influencing cerebral hemodynamics. Furthermore, RCTs involving eTNS and acoustic‐electric TNS have highlighted a possible direction for its application as a novel therapy for DoC; the findings appear to suggest potential clinical relevance, although current evidence remains constrained by small samples, patient heterogeneity, variable stimulation protocols, and limited long‐term follow‐up. At present, trigeminal nerve stimulation for DoC still faces a number of unresolved issues, including uncertainty in the selection of stimulation targets and parameters, an insufficiency of long‐term safety and efficacy data, incompletely characterized mechanisms of action, and a lack of adequately validated preclinical models. Addressing these challenges should be regarded as a high priority in future research.

## Author Contributions

**Haodong Niu:** conceptualization, methodology, validation, formal analysis, investigation, writing – original draft, writing – review and editing. **Yilin Zhang:** formal analysis, investigation, visualization. **Nan Wang**, **Xiaoke Chai**, **Yiyi Zhai**, and **Zhiyao Zheng:** methodology, formal analysis, data curation. **Yangxi Xu**, **Yitong Jia**, and **Kefei Wan:** methodology, validation, formal analysis, writing – review and editing. **Sipeng Zhu**, **Tianqing Cao**, and **Qiheng He:** writing – review and editing. **Minghao Liu**, **Zhuoyang Li**, and **Yichen Wang:** resources, formal analysis, writing – review and editing. **Yi Yang**, **Haitao Huang**, and **Juanning Si:** writing – review and editing, project administration, funding acquisition. **Jizong Zhao:** conceptualization, software.

## Funding

This work was supported by Natural Science Foundation of Beijing Municipality (7252004), International (Hong Kong, Macao, and Taiwan) Science and Technology Cooperation Project (Z221100002722014), Chinese Institute for Brain Research Youth Scholar Program (2022‐NKX‐XM‐02), Science and Technology Innovation 2030 (2022ZD0205300), National High Level Hospital Clinical Research Funding (2025‐PUMCH‐D‐004), National Natural Science Foundation of China (82371197 and 82501457), Joint plan of Liaoning Province Science and technology program (2025JH2/101800242).

## Ethics Statement

The present study is a review that synthesizes information from previously published studies. As such, it does not involve any original research involving human subjects or animals. Therefore, ethical approval and informed consent are not applicable to this manuscript.

## Conflicts of Interest

The authors declare no conflicts of interest.

## Data Availability

The data that support the findings of this study are available from the corresponding author upon reasonable request.
